# Effect of Phenolic Particles on Mechanical and Thermal Conductivity of Foamed Sulphoaluminate Cement-Based Materials

**DOI:** 10.3390/ma12213596

**Published:** 2019-11-01

**Authors:** Xiuzhi Zhang, Qing Yang, Qinfei Li, Heng Chen, Guofa Zheng, Xin Cheng

**Affiliations:** 1 College of Materials Science and Engineering, University of Jinan, Jinan 250022, Chinahitqfli@gmail.com (Q.L.); mse_chenh@ujn.edu.cn (H.C.); 201921100151@mail.ujn.edu.cn (G.Z.);; 2 Shandong Provincial Key Laboratory of Preparation and Measurement of Building Material, Jinan 250022, China

**Keywords:** foamed concrete, dry density, water absorption, compressive strength, thermal conductivity, micro-CT

## Abstract

Foamed concrete materials based on sulpoaluminate cement were prepared by the chemical foaming method. The effects of water–cement ratio, foaming agent, and foaming stabilizer on the mechanical and thermal properties of foamed concrete were studied. Meanwhile, a portion of cement was replaced with foamed phenolic particles to further optimize the performance of foamed concrete; the results show that when the water–cement ratio was 0.53, the foaming agent content was 5%, the foam stabilizer was 1%, and the substitution of phenolic particles was 20%, the performance indexes of foamed concrete were the best. Methods, describing briefly the main methods or treatments applied: dry density was 278.4 kg/m^3^, water absorption was 19.9%, compressive strength was 3.01 MPa, and thermal conductivity was 0.072 W/(m·K). By the pore structure analysis of the foamed concrete suing Micro-CT, it was found that when the replacement amount of phenolic particles was 20%, the pore size of foamed concrete was relatively uniform, the minimum D_90_ was 225 μm respectively. The combination of organic and inorganic matrix and optimized pore structure improved the performance of foamed concrete.

## 1. Introduction

As a cement based thermal insulation material, foamed concrete is more attractive than polymer foam materials for its unique set of properties: it has high heat capacity, excellent fire resistance, and low cost [1,2,3,4,5,6]. At present, most research efforts to foamed cementitious materials have focused on ordinary Portland cement, which has a slow hydration speed at normal conditions and a slow growth rate. Generally, foamed concrete can be demoulded after 24 h under the condition of adding accelerator [7,8,9,10]. Sulphoaluminate cement was applied in our research due to its high hydration rate, high early strength, and strong corrosion resistance. It was reported that material can be demoulded after curing at room temperature for 8 h [11].

Compared with organic thermal insulation materials, the thermal conductivity of conventional cement-based foam materials is higher. The researchers found that the thermal conductivity of organic–inorganic lightweight composite materials can be significantly reduced by using organic foam particles as lightweight aggregates [12,13], the lower thermal conductivity of 0.0848 W/(m·K) was observed in specimens with 82% EPS while this value was 2.5 times higher in samples with 28% EPS volume. Generally, high-strength thermal insulation materials prepared by combining inorganic and organic materials are currently a trend. However, a lower fire resistance was obtained for specimens with higher EPS volume as a result of the fact that EPS particles shrank and lost their strength when subjected to temperature [14]. Phenolic material has good acid and alkali resistance, mechanical properties, heat resistance, and flame retardancy, and the raw material for synthesizing phenolic resin is inexpensive, and is a good thermal insulation material [15,16,17]. However, to the best of our knowledge, the use of phenolic material for preparing thermal insulator has been rarely reported.

Thermal insulating ability of foamed cement composites was significantly determined by its pore structure [18]. Li T. [19] found that the porosity of the material is inversely proportional to the thermal conductivity. As the porosity of the material increases from 60% to 87%, the thermal conductivity decreases from 0.35 W/(m·K) to 0.13 W/(m·K). However, a closed, uniform, fine pore structure is more advantageous for the reduction of thermal conductivity when the porosity is not much different [11,20].

In this study, the foamed concrete was prepared by using sulphoaluminate cement as the cementing material, and the effects of water–cement ratio, foaming agent dosage, and foam stabilizer dosage on its performance were analyzed, and then the mixture ratio was optimized. Based on the optimized mix ratio, the influence of the substitution amount of phenolic particles on the properties of foamed concrete was studied. The pore structure of foamed concrete with excellent properties such as low density and thermal conductivity was analyzed by micro-CT technology, and the mechanism of phenolic particles modified foamed concrete was discussed.

## 2. Materials and Methods

### 2.1. Materials

Sulphoaluminate cement (SAC42.5) was used in this experiment. Its physical properties were measured and the results were shown in Table 1, which complies with the Chinese standard of GB/T 20472-2006. Chemical composition of SAC42.5 was analyzed by using X-ray Fluorescence Spectrometer (Tiger S8, Bruker, Billerica, MA, USA), as presented in Table 2. Hydrogen peroxide solution (H_2_O_2_) with concentration of 30% by mass was selected as the foaming agent. Phenolic particles (Fujian Tianli High-tech Material Co., Ltd., Longyan, Fujian, China) with a fineness such that the 75 µm sieve residue is less than 5%, a water absorption ratio of 80–100%, and a density of about 40 kg/m^3^ were used as cement substitutes.

### 2.2. Preparation of Foamed Concrete

**Figure 1:**
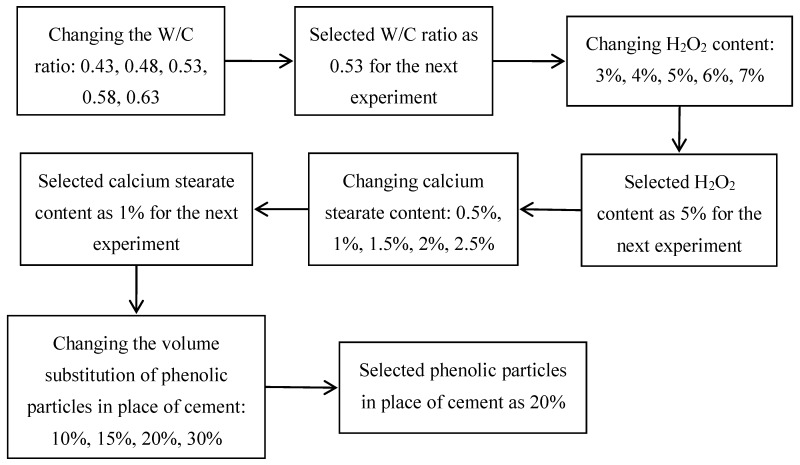
Foamed concrete proportioning design. Note: H_2_O_2_ content/calcium stearate content refers to H_2_O_2_/calcium stearate mass percentage of cement mass.

According to the foamed concrete proportioning design of Figure 1, the procedure of sample preparation is described as follows. (1) All raw materials were weighed accurately, then the cement and other powders were poured into a mixer. (2) Stirring the materials for 120 s at the speed of 140 ± 5 r/min to obtain the powder mixture. (3) After adding water, the cement pastes were obtained by stirring 120 s at speed of 285 ± 10 r/min with a mixer, and then adding H_2_O_2_, continually stirring the mixed material pastes for 120 s at the speed of 285 ± 10 r/min, the foamed cement pastes were obtained. (4) The foamed cement slurry was immediately poured into molds. The exposed top surface was covered with polyethylene film to prevent the water evaporation. (5) They were placed in a standard curing room, and, after 1 d, the foamed concrete was demoulded and then cured under standard condition (20 ± 1 °C, RH ≥ 95%).

### 2.3. Test Methods

#### 2.3.1. Dry Density Measurement

The length, height, and width of 4 × 4 × 16 cm^3^ specimens were measured, and then the volume of specimens was calculated. Meanwhile, the weight of the specimens was tested, thus dry density (*ρ_A_*) can be obtained by Equation (1):

(1) $$\rho_{A}=\frac{m}{v}$$

where *m* is the weight of the specimen after drying, and *v* is the volume of the specimen.

The average dry density of six specimens with the same mix proportion was chosen as the dry density of the foamed concrete.

#### 2.3.2. Compressive Strength Measurement

Calculated the compressive area of the specimen, and then loaded the specimen continuously and uniformly at (2.0 ± 0.5) kN/s speed with a pressure testing machine until the failure occurred, and recorded the failure load. Compressive strength (*R*) was calculated by Equation (2):

(2) $$R=\frac{F}{A}$$

where *F* is the failure load of the specimen, *A* is the compressive area.

#### 2.3.3. Water Absorption Measurement

After cooling the dried samples to room temperature, they were put into a constant temperature water tank with water temperature of (20 ± 5) °C, and then water was added to 1/3 of the specimen height to keep for 24 h. Water was added to 2/3 of the height of the samples. After 24 h, the water was 30 mm higher than the specimen and remained 24 h. Removed the specimen from the water, wiped off the surface moisture with a damp cloth, and immediately weighed each sample (*m_g_*). The water absorption (*W_R_*) was calculated according to Equation (3):

(3) $$W_{R}=\frac{m_{g}-m}{m}$$

where *m* is the weight of the specimen after drying, *m_g_* is the weight of the specimen after water absorption.

#### 2.3.4. Thermal Conductivity Measurement

The thermal conductivity of the specimen was measured by an intelligent double-plate thermal conductivity measuring instrument (produced by Tianjin Yingbeier Technology Development Co., Ltd., Tianjin, China; instrument model: IMDRY3001). The specimen size was 3 × 30 × 30 cm^3^, the preheating time and the measuring time were 30 min and 180 min respectively, and the cold plate and hot plate temperatures were 15 °C and 35 °C, respectively. The experimental data was recorded after the test was completed.

#### 2.3.5. Fluidity of the Cement Pastes

Cement pastes were prepared with various water/cement ratios (W/C = 0.43, 0.48, 0.53, 0.58, 0.63). The initial fluidity was measured with a truncated cone mold (height: 60 mm; top diameter: 36 mm; bottom diameter: 60 mm) in accordance with the Chinese standard of GB/T 8077-2012. The truncated cone placed on a glassy plate was filled with fresh pastes, which then was lifted upward. Allow the fresh pastes flows freely for 30 s, then the maximum diameter of the spread sample and the maximum width perpendicular to that diameter were measured. The average of these two results was defined as the fluidity value.

#### 2.3.6. Micro-X-ray Computed Tomography (Micro-CT) Measurement

Samples with initial dimensions of 4 × 4 × 16 cm^3^ were cut into smaller specimens with the size of 2 × 2 × 2 cm^3^, in order to measure its pore structure, the ZEISS X-CT (model: Xradia 510 Versa, Zeiss, Oberkochen, Germany) was used to detect the middle part of the volume position of the samples, with a pixel size of 20 μm, then 2D and 3D images of the sample were obtained.

## 3. Results

### 3.1. Effect of Water–Cement Ratio on Properties of Foamed Concrete

The performance of foamed concrete was significantly determined by the water to cement ratio, which can influence the fluidity behavior and pore structures of foamed composites, thus is able to affect its mechanical strength property and thermal conductivity value. In order to observe the effect of water–cement ratio on these properties of foamed cement-based materials, cement-based composites with different water–cement ratios varying from 0.43 to 0.63 were prepared, H_2_O_2_ as foaming agent was incorporated as well in the amount of 5%, by cement mass.

**Figure 2:**
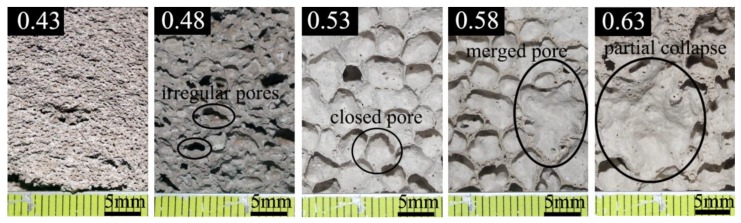
Surface morphology of specimens with different water–cement ratios.

**Figure 3:**
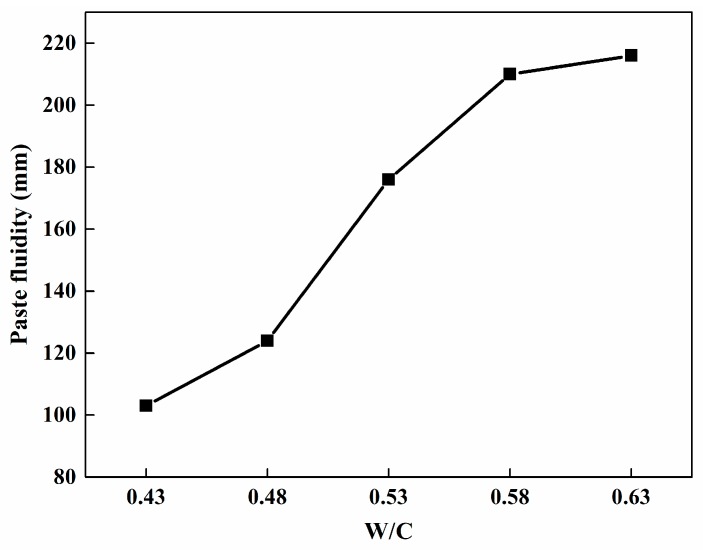
Effect of water–cement ratio on pastes fluidity.

Figure 1 shows the surface morphology of foamed sulphoaluminate cement-based composites with varying water–cement ratios which were obtained by a digital camera (Canon: EOS 600D). It can be apparently observed that the amount of mixing water resulted in enormous effects on pore property of foamed composites. When the W/C ratio was 0.43, normal foaming is not possible. When a relative lower W/C ratio of 0.48 was used, irregular fine pores were embedded into cement samples. With the increase in the mixing water, in general the shape of pores tends to performance as a sphere, and the size of pores grows larger and larger. For the samples with a W/C ratio of 0.53, foamed composites were found to be involved with well distributed bubbles with a larger size than that of a W/C ratio of 0.48, the maximum diameter of the pores was 5 mm. Distinctive pore structure was discovered in those samples with W/C ratios of 0.58 and 0.63, more merged pores lead to poor cement structure which is mainly caused by the combination of large pores, as demonstrated in Figure 2, the maximum pore size was approximately 15 mm. The reasons for different pore structures were associated with the viscosity of the cement pastes which affects the foaming ability of H_2_O_2_. The viscosity property of the pastes was closely related to the fluidity behavior, and the relationship between the fluidity of the pastes and W/C ratio is presented in Figure 3. It can be seen that the fluidity of the cement pastes increase with W/C ratio, indicating that the viscosity of the pastes decreases with W/C ratio. However, it should be noted that an appropriate viscosity is needed to make foamed concrete. When the W/C ratio was less than 0.53, the cement pastes had a relatively small fluidity and large viscosity, the gas expansion force cannot break through the resistance from the cement pastes, resulting in a poor pore structure in Figure 2. When W/C ratios were greater than 0.53, the fluidity of the cement pastes is too large, meaning that the pastes cannot wrap the gas and the pores merge with each other. Therefore, the proper fluidity can make the O_2_ produced by decomposition of H_2_O_2_ expand normally in the cement pastes to produce pores with even size and distribution. In this study the best W/C ratio would be 0.53, based on Figure 2.

**Figure 4:**
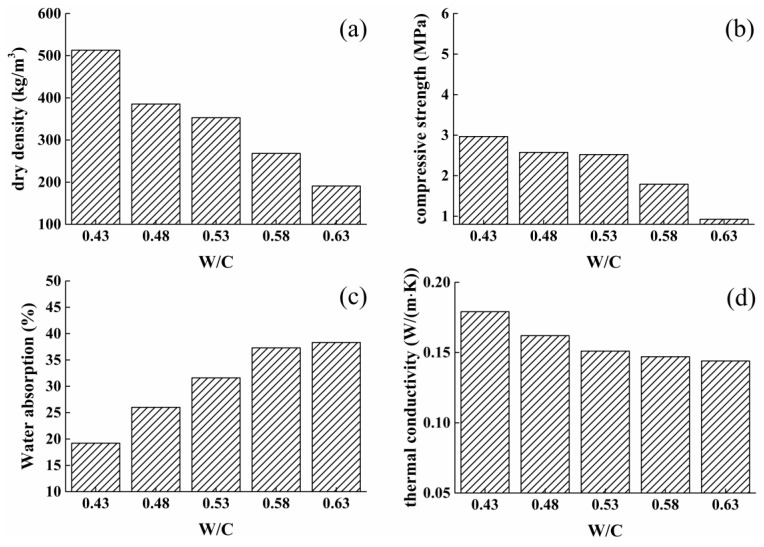
Effect of water-cement ratio on properties of foamed concrete: (**a**) dry density; (**b**) compressive strength; (**c**) water absorption; (**d**) thermal conductivity.

For further exploring the corresponding relationship between mixing water and pore structure of foamed cementitious based materials, the dry density value, water absorption ability, compressive strength, and thermal conductivity properties of foamed composites were measured and presented in Figure 4. First of all, the volume content of pores inside samples can be well depicted by its dry density value, as demonstrated in Figure 4a. It can be observed that dry density value was significantly determined by the mixing water of samples and W/C ratios of 0.43, 0.48, 0.53, 0.58, and 0.63 are used, the dry density value of composites are about 513.2, 385.3, 352.8, 268.1 and 190.9 kg/m^3^, respectively. The increasing porosity of composites influenced by adding more water could account for the decreasing tendency of dry density value. The pore content of composites can also govern the strength property as shown in Figure 4b. It was revealed that the compressive strength is relatively similar when W/C ratios of 0.43, 0.48, and 0.53 are used, strength values are 2.967, 2.574, and 2.521 MPa, respectively. When more water was added, a sharp decrease in strength was found; strength values of samples decreased to 1.789 and 0.923 MPa with W/C ratios of 0.58 and 0.63. The decreasing compressive strength of foamed concrete was absolutely attributed to the increasing porosity, the result is consistent with the dry density result. Furthermore, pores embedded in foamed composites exist in two types, merged pores and closed pores [21]. Merged pores are filled with air and allow liquid water to pass through, while closed pore cannot transport moisture and air. Thus closed pores embedded into composites are beneficial for the thermal conductivity of foamed concrete. However, to the best of our knowledge, it is very difficult to distinguish between merged and closed pores inside cement. In our research, we measured the water absorption ability of foamed composites to explore the amount of merged pores, as illustrated in Figure 4c. It was obtained that with higher W/C ratio, water absorption of samples apparently increases, which shows a higher amount of merged pores were embedded into the cement system, due to higher fluidity of cement pastes with a higher W/C ratio. Moreover, Figure 4d presents thermal conductivity properties of foamed concrete and it was found that the conductivity values decreased sharply with increasing mixing water in cement, especially when W/C ratios vary from 0.43 to 0.53, due to the sharp increase in porosity of samples. With the further extension of mixing water, although a higher amount of pores was embedded inside composites, no obvious decrease in conductivity values was found. The increasing percentage of merged pores makes the thermal conductivity of the samples of with high W/C ratios increase due to the path complexity of flowing air. Combined with the strength result, therefore, it was concluded that 0.53 is the optimum W/C ratio for meeting a low thermal conductivity value and the satisfied compressive strength property, so in the next experiment, the fixed W/C ratio was 0.53.

### 3.2. Effect of the Dosage of Foaming Agent on Properties of Foamed Concrete

The amount of foaming agent added into cement pastes should be considered due to its enormous effect on the pores structure of system. With the purpose of exploring the optimizing dosage of H_2_O_2_, foamed sulphoaluminate cement composites with W/C ratio of 0.53 were prepared with varying added amount of H_2_O_2_ (3%, 4%, 5%, 6%, 7%) and their dry density, compressive strength, water absorption, and thermal conductivity were both measured.

**Figure 5:**
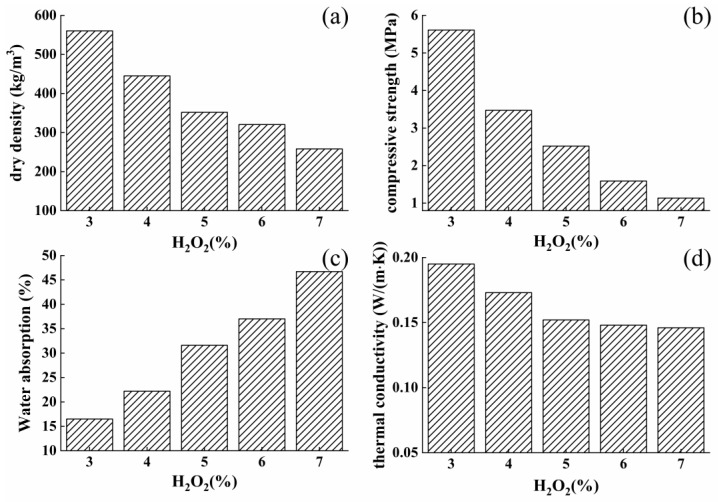
Effect of H_2_O_2_ content on properties of foamed concrete: (**a**) dry density; (**b**) compressive strength; (**c**) water absorption; (**d**) thermal conductivity.

To begin with, Figure 5a,b show the dry density and compressive strength of foamed composites respectively. It was found that dry density property of cement-based materials with high porosity largely decreased with the addition of H_2_O_2_, dry density values with 3%, 4%, 5%, 6%, and 7% are about 560.4, 445.1, 351.8, 320.5, and 258.2 kg/m^3^, respectively. The result demonstrates that a larger amount of bubbles was produced and then embedded into the cement system due to higher added dosage of H_2_O_2_. Compressive strength performance of composites was also governed by its pore content and it was illustrated from Figure 5b that the strength property of foamed materials shows a sharp decrease with the increasing pore content of composites. The compressive strength values decreased by 55%, from about 5.6 to 2.52 MPa, with the dosage of H_2_O_2_ enhanced from 3% to 5%. When a higher amount of foaming agent was added, overly large amounts of large bubbles were generated in the system, leading to an obvious volume shrinkage of system, especially with H_2_O_2_ dosage of 7%, when cement pastes cannot support bubbles in large sizes. Therefore, the poor structure of composites with 6% and 7% H_2_O_2_ exhibit extremely low compressive strength, with values of about 1.59 and 1.13 MPa, respectively. Additionally, the effect of foaming agent on the amount of merged pores was measured by testing the water absorption ability of composites, which was drawn in Figure 5c. The experimental data demonstrates an enhancement in absorbing water ability, absorbing rates of samples change from 16.5% to 46.7%, thus resulting in a conclusion that higher addition of foaming admixture can produce much more quantity of merged pores inside cement matrix. Finally, thermal conductivity property of foamed composites, governed by the amount of internal closed pores, was considered. The experimental results are shown in Figure 5d. It was observed that thermal conductivity values show a trend to decrease, conductivity values of samples with 3%, 4%, 5%, 6%, and 7% are around 0.195, 0.173, 0.152, 0.148, and 0.146 W/(m·K). It is worthy noticing that thermal conductivity property was obviously enhanced with less than 5%, while more addition bubbles cannot cause an evident effect on thermal transfer ability. Therefore, balance in the consideration of cement strength and thermal insulating ability of foamed composites, 5% H_2_O_2_ was chosen as the optimizing dosage for preparing foamed samples, and was used in the following experiment.

### 3.3. Effect of Calcium Stearate on Properties of Foamed Concrete

Besides the viscosity of pastes and amount of embedded bubbles, the foam stability during samples preparation is of significance to samples properties. It has been reported by Liu P. [4] that calcium stearate as surfactant can adsorb on the surface of cement particles to make them hydrophobic, and then adsorbs on the gas–liquid interface of bubbles to stabilize the foam. Therefore, calcium stearate was mixed with sulphoaluminate cement particles to stabilize internal bubbles with the amounts of 0%, 0.5%, 1%, 1.5%, 2%, and 2.5%, with W/C ratios of 0.53 and 5% H_2_O_2_.

**Figure 6:**
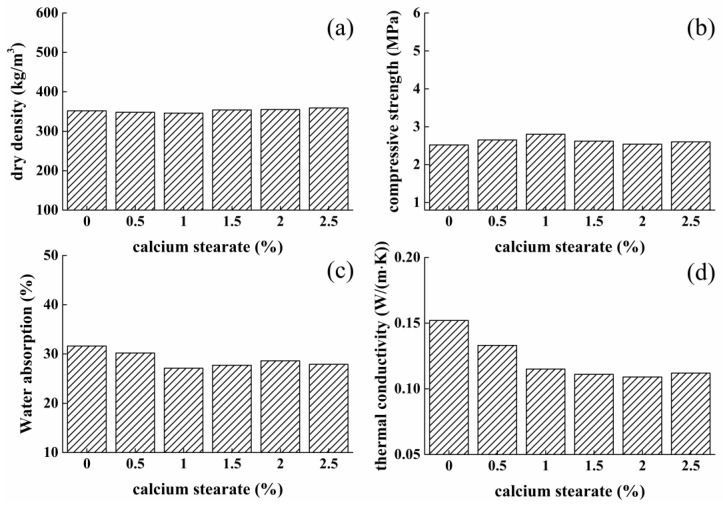
Effect of calcium stearate content on properties of foamed concrete: (**a**) dry density; (**b**) compressive strength; (**c**) water absorption; (**d**) thermal conductivity.

**Figure 7:**
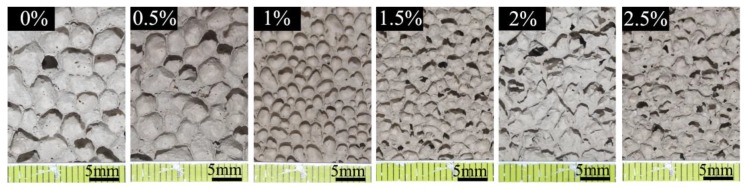
Surface morphology of specimens with different calcium stearate.

Figure 6a,b present dry density and compressive strength property of foamed materials with varying calcium stearate dosage. It was demonstrated that the dry density and compressive strength was little influenced by calcium stearate. However, with the incorporation of calcium stearate, water absorption tests showed that the amount of merged pores decreased with dosage of 1%, as illustrated in Figure 6c. It can also be clearly seen from Figure 7 that as the amount of calcium stearate was increased from 0% to 1%, the merged pores of the sample were reduced, the pore diameter was decreased, and the pore walls were thickened. Furthermore, it was found from thermal conductivity measurements shown in Figure 6d that thermal insulating ability of foamed composites was enhanced by using calcium stearate, this is because the amount of closed pores are increased significantly. The amount of calcium stearate above 1% does not result in the obvious improvement in the water absorption and conductivity value, and the values fluctuated slightly, at this time, the pore structure was obviously not as good as 1% (Figure 7). Therefore, 1% amount of calcium stearate was added in our research.

### 3.4. Effect of Phenolic Particles on Properties of Foamed Concrete

Incorporating organic thermal insulation materials with inorganic thermal insulators has brought broad interests, with the purpose of enhancing the thermal insulating property. In this research, foamed phenolic materials, as organic insulator, has been incorporated with sulphoaluminate cement-based composites due to its good thermal insulating ability, low density, high porosity, excellent alkali resistance, good fire resistance, low cost, and being commercially available [22,23,24]. But, to the best of our knowledge, the incorporation of phenolic with sulphoaluminate cement-based materials has been very little reported as yet. For exploring the effect of phenolic particles on thermal conductivity performance of cement-based materials, sulphoaluminate cement powders were partially replaced by phenolic particles by the amount of 0%, 10%, 15%, 20%, and 30% to prepare composites, and the optimum W/C ratio, dosage of H_2_O_2_, and calcium stearate were used.

**Figure 8:**
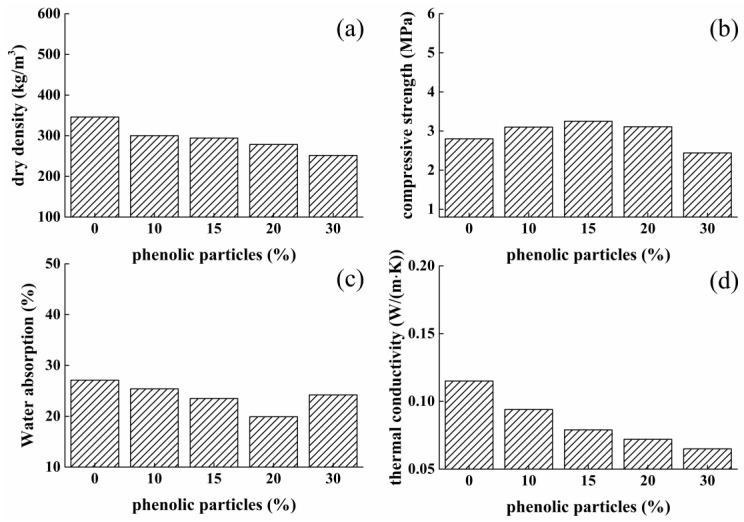
Effect of phenolic particles content on properties of foamed concrete: (**a**) dry density; (**b**) compressive strength; (**c**) water absorption; (**d**) thermal conductivity.

Total content of pores inside the phenolic–cement system was indirectly characterized by the dry density value of samples, as plotted in Figure 8a. It was shown that the replacement by phenolic powders contributes to a slight decrease in the dry density value of composites, that provides a fact that the total amount of pores was slightly increased. Dry density values decrease from about 346 to 251.6 kg/m^3^, with 30% cement powders were replaced. This is because phenolic particles have very low density. However, it was found from the strength result (as shown in Figure 8b) that with increasing amount of pores embedded, the compressive strength of composites increases firstly and then shows a trend to decease. Foamed cement samples with replacement of 15% phenolic particles exhibit the highest strength, 3.25 MPa, 16% higher than control samples of 2.8 MPa.

**Figure 9:**
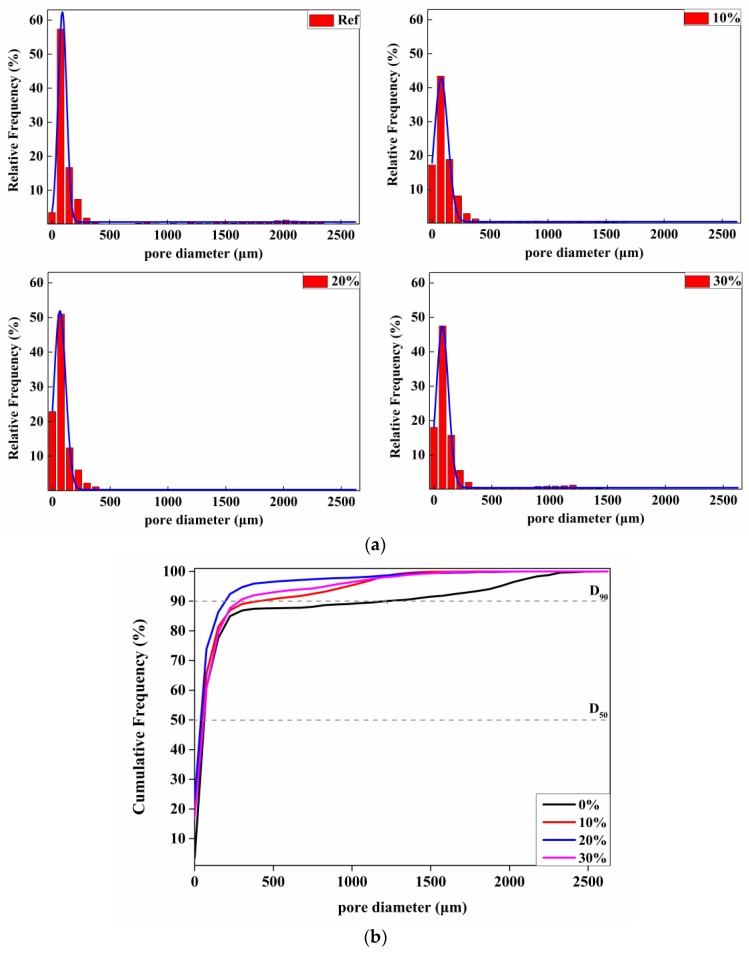
Pore size analysis: (**a**) pore size distributions of the foamed concretes; (**b**) cumulative frequency distributions of pore size.

The disagreement between dry density and strength results is mainly attributed to the size distribution of pores. Void analysis of foamed composites partially replaced by phenolic particles was measured by micro-CT technology, experimental result was plotted in Figure 9. Void analysis shows that the diameter of embedded pores of every sample primarily ranges in 75–150 μm. A distinct phenomenon is worthy noticing that with the replacement of phenolic powders, the amount of smaller pores with diameter less than 75 μm in foamed matrix was significantly increased, 7.21%, 12.18%, 15.01%, 20.28%, 16.49%, which proves the size of generated pores becomes much finer due to the addition of phenolic powders, resulting in the enhancement in strength of samples with the replacing rate of less than 15%. One more thing required to note is that water absorbing ability of samples was decreased when adding phenolic powders up to 20% (Figure 8c). The reason behind this phenomenon is that after mixing with phenolic powders, large pores with a diameter of more than 1500 μm generally disappear, especially in samples with 20% phenolic particles, most internal pores are in the diameter of less than 500 μm, as shown in Figure 9a, also, it can be obtained from Figure 8b that the D_90_ of the material has minimum values of 225 μm (D_90_ represents the pore diameters when the cumulative pore distribution is 90%). Results shows that the amount of merged pores are decreasing and the pores distribution is becoming more uniform, especially in composites with 20% phenolic particles.

Thermal conductivity value of foamed phenolic–cement composites was obtained for the characterization of its thermal insulating ability. Experiment result were shown in Figure 8d and it was illustrated that thermal insulating property was largely enhanced with the increasing proportion of closed pores and decreasing amount of merged pores inside the cement matrix, which was consistent with pore analysis. With the replacements of 0%, 10%, 15%, 20%, and 30%, conductivity values of composites are about 0.115, 0.094, 0.079, 0.072, and 0.065 W/(m·K), respectively. Therefore, the incorporation of phenolic materials plays a significant role in realizing a low thermal conductivity value and thus provides a good chance serving as an excellent thermal insulator. Considering the replacement of 30% cement powders, samples presented very poor structure and thus poor compressive strength due to the sharp decrease in viscosity of pastes. Therefore, we selected the replacement of 20% as the optimum dosage.

**Figure 10:**
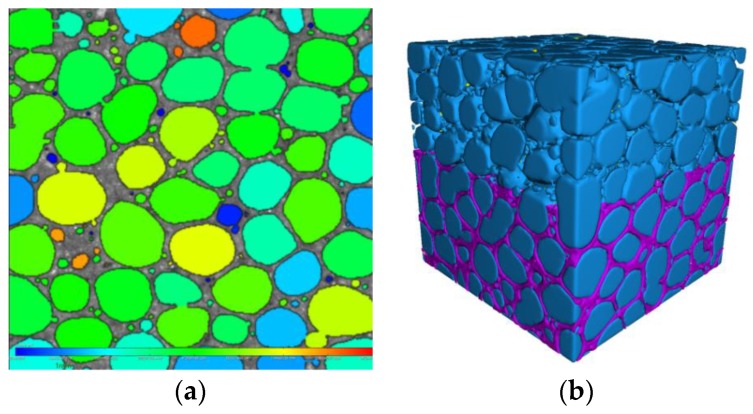
Micro-CT reconstruction graph: (**a**) 2D structure diagram; (**b**) 3D structure diagram.

The 2D rendering map (Figure 8a) shows that the pore distribution of the material is relatively uniform, and there is almost no pore (red). Figure 10b represents the pore network structure of 3D, in which the pores account for 87% (blue) and the cement matrix accounts for 25% (purple).

## 4. Mechanism Analysis of Phenolic-Foamed Concrete 

**Figure 11:**
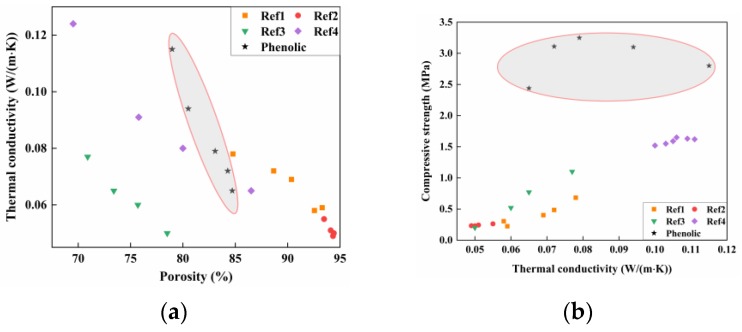
Mechanism analysis of phenolic-foamed concrete: (**a**) the relationship between porosity and thermal conductivity; (**b**) the relationship between thermal conductivity and compressive strength.

For better evaluating the enhancement of phenolic powders in thermal conductivity property of cement-based materials, we summarized many of experimental results in several previous research [2,11,25], the shaded part is the result of this experiment. Figure 11a presented the thermal conductivity behavior and porosity property of foamed cementitious materials, it can be found that the decrease in thermal conductivity is mainly achieved by greatly increasing the porosity. Figure 11b presented the relationship between thermal conductivity values and compressive strength of samples, that is, the decrease in thermal conductivity is accompanied by the decrease in compressive strength. The appropriate amount of phenolic particles can significantly reduce the thermal conductivity of foamed concrete with little change in porosity, and the compressive strength of foamed concrete is basically stable at about 3.0 MPa. There are several possible reasons for this: (1) phenolic particles together with cement pastes form a foamed concrete skeleton, the thermal conductivity of phenolic particles at room temperature is 0.02–0.03 W/(m·K), which is close to the thermal conductivity of air (0.023 W/(m·K)), which is much smaller than the thermal conductivity of cement (about 0.67 W/(m·K)). Moreover, the combination of organic and inorganic reduces the thermal conductivity of the foamed concrete skeleton; (2) combining the properties of foamed concrete (Figure 8) with micro-CT analysis (Figure 9), it can be seen that when the substitution of phenolic particles is 20%, the thermal conductivity of foamed concrete is 0.072 W/(m·K), which is less than the theoretical value of 0.106 W/(m·K). It is indicated that the organic–inorganic phase composite optimizes the pore structure of the foamed concrete, increases the number of closed pores, refines the pore size, and concentrates the pores. The optimization of these micro-structures is beneficial to the improvement of various properties of the foamed concrete; (3) in addition, the improvement of the pore structure of the foamed concrete makes the heat transfer path of the solid phase heat conduction longer and the heat transfer direction becomes tortuous, so that the heat transfer speed is greatly slowed down.

## 5. Conclusions

Based on the results of this investigation, the following can be concluded:

(1) The increase in the water–cement ratio and the increase in the amount of the foaming agent will reduce the dry density and compressive strength of the foamed concrete-based material, and the proper amount of the foam stabilizer can effectively stabilize the bubbles, prevent the bubbles from being combined and broken, and reduce the water absorption rate.
(2) When the water–cement ratio is 0.53, the foaming agent dosage is 4–5%, and the foam stabilizer is 1%, the cement slurry is easy to mix, the pore distribution is relatively uniform, and there is no slurry delamination and collapse phenomenon.
(3) The use of phenolic particles instead of cement to prepare foamed concrete not only changes the thermal conductivity of the substrate but also optimizes the pore structure, and the compressive strength is stable while greatly reducing the thermal conductivity. When the substitution of phenolic particles is 20%, the dry density of the foamed concrete reaches the grade B03, and the compressive strength grade reaches B05 [26].

## Figures and Tables

**Table 1:** Physical properties of sulfoaluminate cement (SAC).

Material	Specific Surface Area (m^2^/kg)	Standard Consistency (%)	Setting Time (min)		Flexural Strength (MPa)			Compressive Strength (MPa)		
			Initial	Final	1 d	3 d	28 d	1 d	3 d	28 d
SAC	364	27.7	15	20	5.1	6.1	7.3	30.9	42.7	54.8

**Table 2:** Chemical composition of sulfoaluminate cement (SAC).

Oxides Chemical Composition	SiO_2_	CaO	Al_2_O_3_	Fe_2_O_3_	MgO	K_2_O	Na_2_O	TiO_2_	SO_3_	LOI
Wt (%)	9.60	45.16	21.64	2.45	1.28	1.38	0.17	1.03	10.73	6.35
